# Giant Mandibular Ameloblastoma with Rare Hypercalcemia: A Case Report and Literature Review

**DOI:** 10.3390/medicina59111956

**Published:** 2023-11-06

**Authors:** Wenyi Shen, Chenlu Xu, Pan Wang, Junpeng Chen, Dan Yu, Huiyong Zhu

**Affiliations:** 1 Department of Oral and Maxillofacial Surgery, The First Affiliated Hospital, Zhejiang University School of Medicine, Hangzhou 310003, China; wyshen@zju.edu.cn (W.S.); zdxcl@zju.edu.cn (C.X.); wangpp@zju.edu.cn (P.W.); cjp1221@zju.edu.cn (J.C.); 2 Department of Stomatology, Zhejiang University School of Medicine, Hangzhou 310058, China

**Keywords:** ameloblastoma, hypercalcemia, multi-disciplinary teams, surgery, marsupialization, renal impairment

## Abstract

Ameloblastoma is the most common benign odontogenic tumor with local invasion and high recurrence, which generally occurs in the jaw bones. Hypercalcemia is a common paraneoplastic syndrome that is commonly observed in patients with malignancies but rarely encountered in patients with benign tumors. Thus far, not many cases of ameloblastoma with hypercalcemia have been reported, and the pathogenic mechanism has not been studied in depth. This paper presents a case report of a 26-year-old male diagnosed with giant ameloblastoma of the mandible, accompanied by rare hypercalcemia. Additionally, a review of the relevant literature is conducted. This patient initially underwent marsupialization, yet this treatment was not effective, which indicated that the selection of the appropriate operation is of prime importance for improving the prognosis of patients with ameloblastoma. The tumor not only failed to shrink but gradually increased in size, accompanied by multiple complications including hypercalcemia, renal dysfunction, anemia, and cachexia. Due to the contradiction between the necessity of tumor resection and the patient’s poor systemic condition, we implemented a multi-disciplinary team (MDT) meeting to better evaluate this patient’s condition and design an individualized treatment strategy. The patient subsequently received a variety of interventions to improve the general conditions until he could tolerate surgery, and finally underwent the successful resection of giant ameloblastoma and reconstruction with vascularized fibular flap. No tumor recurrence or distance metastasis was observed during 5 years of follow-up. Additionally, the absence of hypercalcemia recurrence was also noted.

## 1. Introduction

Ameloblastoma is a common benign tumor that occurs most frequently in the jawbones, comprising approximately 1% of all oral tumors. In the 5th edition classification of the Head and Neck Tumors published by the World Health Organization (WHO, Geneva, Switzerland), five separate ameloblastoma categories are distinguished: unicystic ameloblastoma, extraosseous/peripheral ameloblastoma, conventional ameloblastoma, adenoid ameloblastoma, and metastasizing ameloblastoma [1]. Surgery is the main treatment modality for ameloblastoma, which can be separated into conservative treatment and radical treatment [2]. Although histologically benign, ameloblastomas exhibit a locally invasive growth pattern and a high recurrence rate. The best choice of surgical treatment for ameloblastomas is currently debated. Conservative treatment can maximize the preservation of normal tissue and reduce maxillofacial deformities, while the recurrence rate after surgery is quite high [3]. The main objective of radical treatment is to complete the removal of the tumor and the marginal tissue at risk for infiltration of tumor cells to minimize recurrence [4,5]. However, this approach leads to severe maxillofacial deformity and results in compromised stomatognathic function, which causes impaired quality of life and psychological distress. Therefore, oral and maxillofacial surgeons are faced with a dilemma between conservative treatment and radical treatment when dealing with patients with ameloblastoma, especially giant ameloblastoma [6]. Some studies have shown that cystic ameloblastoma decreases in size after marsupialization, and the shrinking of tumor bulk makes subsequent surgical treatment easier and less extensive [7,8]. However, not all patients with ameloblastoma benefit from this method, and some even developed severe complications.

In the advanced stage, patients with tumors often suffer from a range of severe complications. Hypercalcemia is a fairly common paraneoplastic syndrome, which is frequently observed in patients with malignant tumors (such as lung cancer, multiple myeloma, breast cancer, and renal cancer), rather than those with benign tumors. However, some cases of ameloblastoma with hypercalcemia have been reported, which suggests that ameloblastoma, a benign tumor, may also cause hypercalcemia via certain mechanisms similar to malignancy. Until now, however, such cases have been extremely rare, as has renal dysfunction from this condition, and the relevant pathogenic mechanisms have not been clearly determined. In addition, many oral and maxillofacial surgeons are challenged when dealing with complex ameloblastoma cases accompanied by many kinds of symptoms and complications.

This paper reports a rare case of giant mandibular ameloblastoma with severe hypercalcemia and provides a review of the relevant literature. The patient’s tumor grew aggressively after marsupialization, accompanied by multiple complications including hypercalcemia, renal dysfunction, cachexia, and anemia. The urgency of tumor resection created a clear contradiction with the patient’s poor physical condition that caused difficulty for him to tolerate the surgery. To better evaluate the patient’s condition and develop a more individualized treatment plan, we implemented an MDT meeting with various clinical departments in our hospital. Subsequently, the patient underwent a series of interventions to improve his general condition, and ultimately successfully received tumor resection and second-stage reconstruction with a vascularized fibular osteomyocutaneous flap, which yielded a great outcome. 

## 2. Case Report

A 26-year-old male was referred to our hospital, who complained of a giant tumor in the right mandible, which had become significantly larger after marsupialization for 3 months with recurrent oral bleeding.

**Figure 1:**
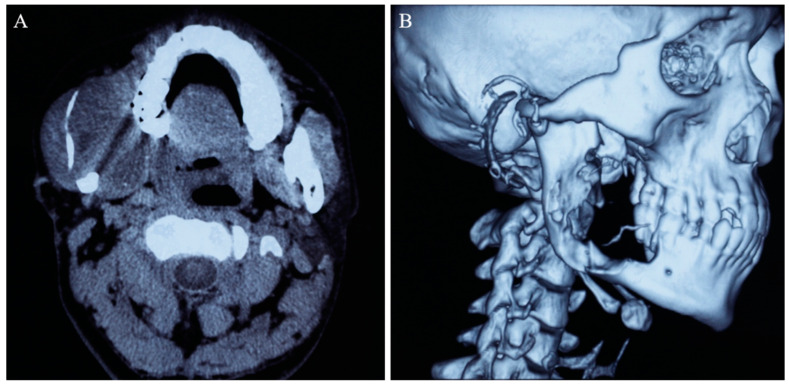
CBCT imaging (**A**) and three-dimensional reconstruction of the skull (**B**) demonstrated extensive bony destruction and soft tissue involvement by the right mandibular tumor.

This patient had been previously diagnosed with ameloblastoma and received marsupialization at the age of 25. He then presented with the initial symptoms of loosening of the right lower wisdom tooth, swelling of the right cheek, and recurrent overflow of dark brown liquid oral discharge. The initial cone beam computed tomography (CBCT) examination revealed a huge unilocular radioluscent lesion in the right mandible, extending from the right mandibular body to the ascending ramus (Figure 1). Cortical-bone destruction in the buccal and lingual regions and knife-edge root resorption of the right lower second molar were observed. Such evidence strongly implicated the diagnosis of ameloblastoma, and eventually this diagnosis was validated by histopathological examination. The patient underwent marsupialization in a local hospital, and the blood examination showed no relevant abnormalities in hematology or biochemistry during hospitalization.

After marsupialization, the patient noted that the tumor was gradually increasing in size, which led to facial deformity, adjacent teeth loosening, restricted mouth opening, and recurrent oral bleeding. The large size of the tumor impeded solid food consumption, leading to severe nutritional deficiencies and progressive weakness. Therefore, the patient visited several hospitals to seek medical treatment. The examination showed a decrease in hemoglobin and an increase in creatinine and total calcium. The patient was diagnosed with iron deficiency anemia and chronic kidney disease, which was likely related to the progressively enlarged ameloblastoma. However, the poor general condition of the patient made him unfit for tumor resection. He received a series of interventions to improve the systemic conditions, including blood transfusion, gastrostomy, and oral beraprost sodium therapy; however, these measures yielded limited efficacy.

**Figure 2:**
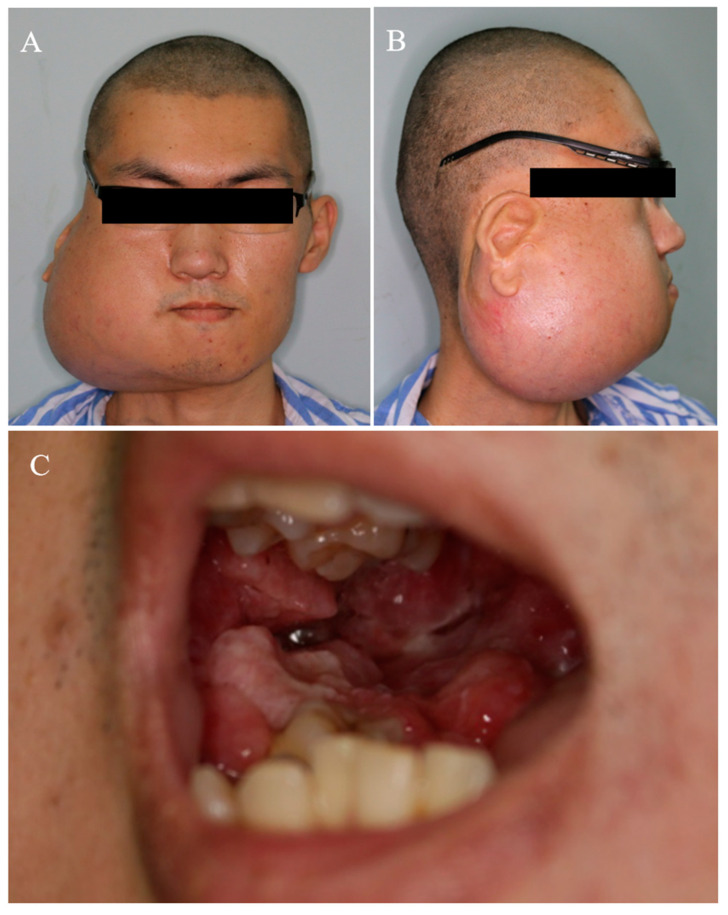
Right mandibular ameloblastoma resulted in huge swelling of mandible and significant facial asymmetry (**A**,**B**). Intraoral examination showed that the right buccal vestibule and gums presented widely swollen with a cauliflower-like mass (**C**).

**Figure 3:**
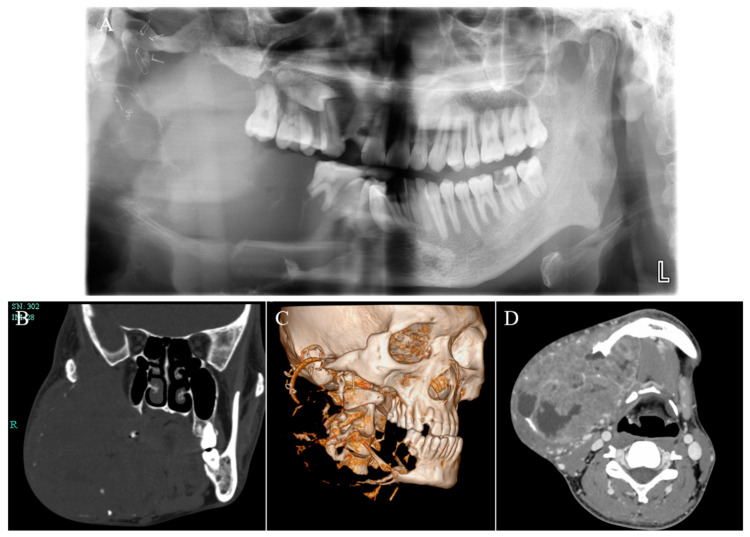
Panoramic radiography (**A**), CT (**B**), three-dimensional reconstruction (**C**), and MRI (**D**) showed an expansive growth pattern of the tumor with bony destruction and huge soft tissue mass.

One year after marsupialization, the patient was referred to our hospital urgently due to his progressively serious condition. During the first physical examination, the patient was malnourished, weak, and exhibited cachexia (BMI = 16). Specialized examination revealed a huge swelling in the right mandible causing significant facial asymmetry (Figure 2A,B). On palpation, the mass exhibited as fixed, ill defined, non-fluctuant, and non-tender. The right buccal vestibule and gums were affected intraorally by this enlargement, appearing widely swollen with a cauliflower-like mass (Figure 2C). This mass was prone to bleeding, and yellow pus was observed when squeezed (infection). There was no cervical lymphadenopathy or symptoms of distant metastases. Panoramic radiography, CBCT, and MRI imaging showed extensive bony destruction of the right mandible with a huge soft tissue mass, measuring 7.3 cm × 9.5 cm (Figure 3). The lesion involved the right cheek muscle and extended upward to the right palatal fossa, and the posterior lateral wall of the right maxillary sinus was also absorbed and destroyed.

Unfortunately, the patient’s systemic condition was poor and complex, which greatly restricted the implementation of tumor resection. Biochemical and blood examination upon the admission showed the elevation of total serum calcium to 3.64 mmol/L (normal range 2.03~2.54 mmol/L), that of serum creatinine to 138 µmol/L (normal range 59~104 µmol/L), the decrease in inorganic phosphorus to 0.71 mmol/L (normal range 0.87~1.45 mmol/L), and that in hemoglobin to 91 g/L (normal range 131~172 g/L). There was a strong contradiction between the urgency of tumor surgery and the physical condition of the patient, which was too poor for him to tolerate surgery. To address this intractable problem, we conducted an MDT meeting to better analyze the patient’s condition, summarize the multi-disciplinary recommendations, and develop an appropriate treatment plan (Table 1). The participating departments included radiology, neurosurgery, nephrology, anesthesiology, endocrinology, and so on. After the joint evaluation of the MDT meeting, the patient was finally diagnosed with right mandibular ameloblastoma associated with hypercalcemia, chronic kidney disease, iron deficiency anemia, and cachexia. Physicians from various departments analyzed the patient’s abnormal biochemical parameters and proposed possible explanations for these abnormalities (Table 2). It is worth noting that the patient’s hypercalcemia could not be ruled out as being related to giant mandibular ameloblastoma.

**Figure 4:**
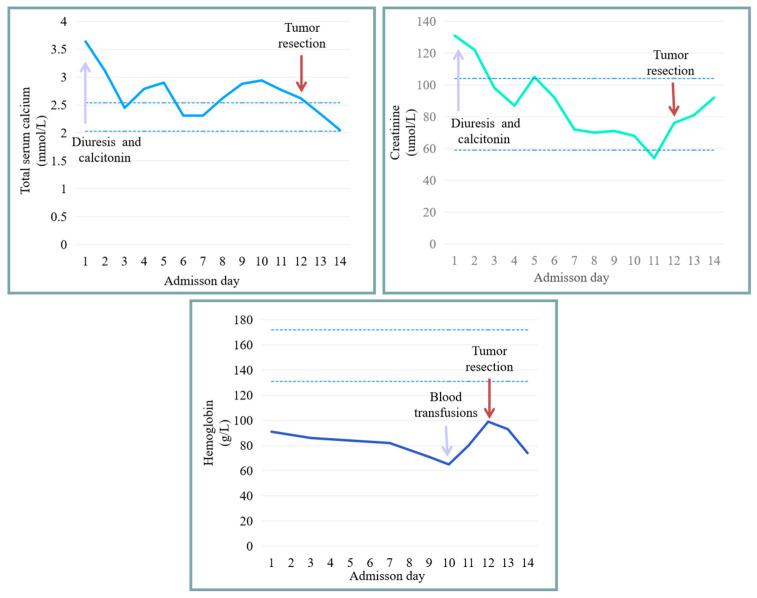
The change in total serum calcium levels, serum creatinine levels, and hemoglobin levels after interventions.

Subsequently, we implemented a series of preoperative interventions to improve the patient’s surgical tolerance, including (1) reducing the blood calcium concentration by diuresis (furosemide, 20 mg, QD or BID) and calcitonin (Miacacic, 100 IU, QD or BID); (2) improving anemia by iron supplementation (Rifeinen, 150 mg, BID), and red blood cell transfusions before operation (400 mL); and (3) improving cachexia by enteral nutrition therapy (oral feeds or via gastrostomy tube). Overall, these interventions yielded good outcomes. The patient’s total serum calcium levels and serum creatinine levels gradually improved and stabilized within relatively normal ranges. The hemoglobin levels tended to improve upon the transfusion of blood products, but repeated oral bleeding and long-term malnutrition would make it difficult for the patient’s hemoglobin levels to restore to the normal range in a short time (Figure 4).

**Figure 5:**
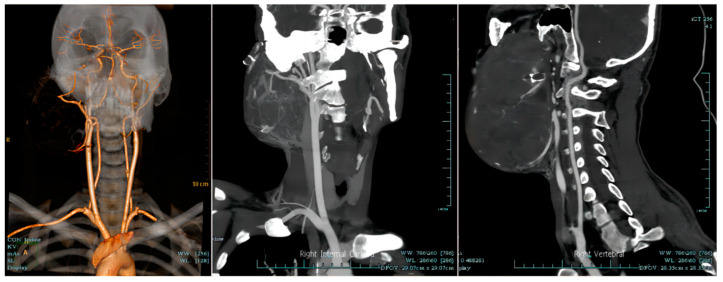
The computerized tomography with angiography of the neck vessels suggested that the right mandibular ameloblastoma was supplied by multiple branches of the right external carotid artery.

**Figure 6:**
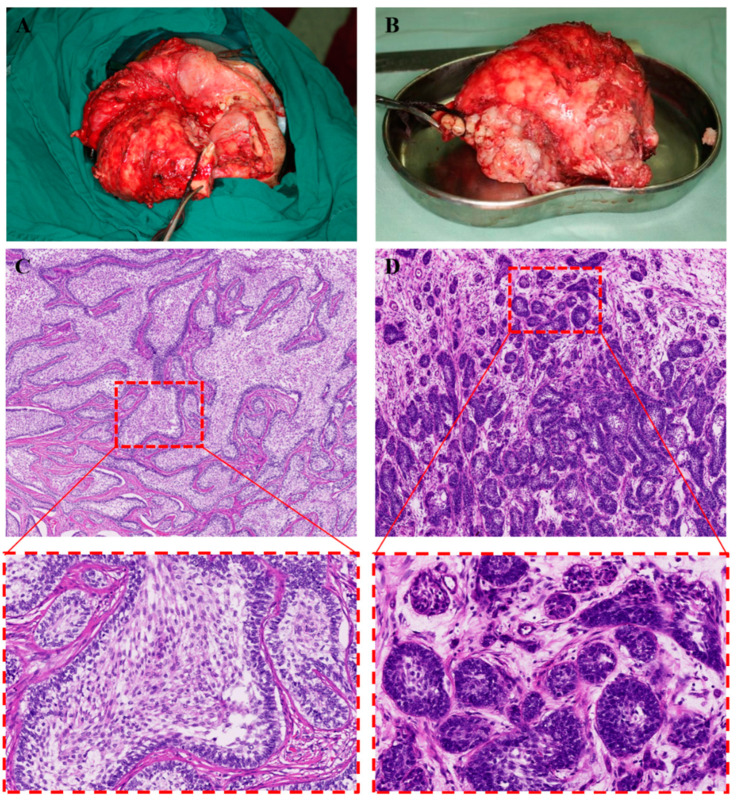
Gross appearance of the ameloblastoma after surgical excision (**A**,**B**). Histological biopsy of the resected specimen showed the ameloblastoma was a mainly plexiform type (**C**) with a small amount of follicular structure (**D**).

**Figure 7:**
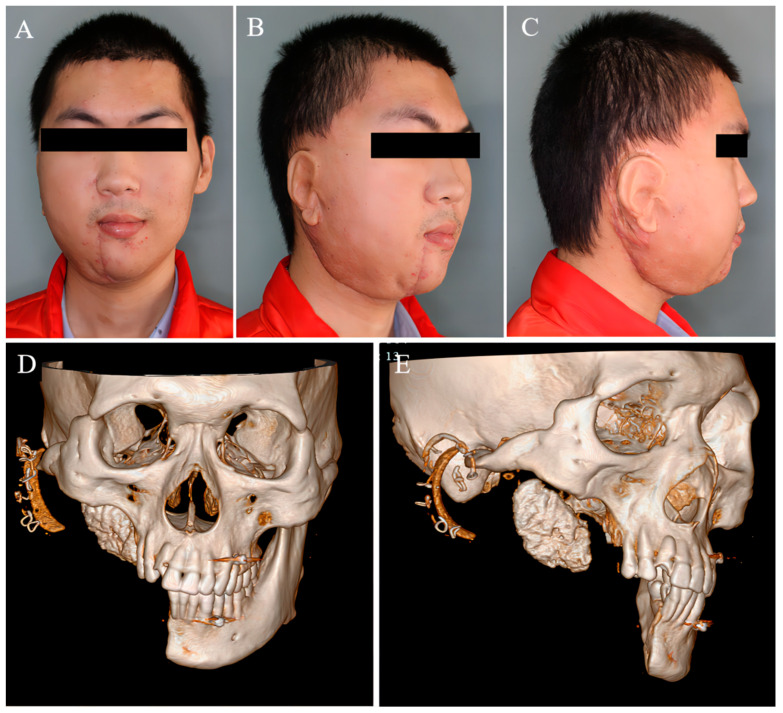
The patient’s facial deformity had improved greatly after tumor resection. (**A**–**C**) Clinical photos and (**D**,**E**) three-dimensional reconstruction.

At 12 days after the interventions, multi-disciplinary experts concluded that the patient’s systemic condition was satisfactory to tolerate tumor resection (total calcium 2.77 mmol/L, creatinine 71 µmol/L, hemoglobin 91 g/L). The preoperative computerized tomography with angiography (CTA) of the neck vessels suggested that the tumor was supplied by multiple branches of the right external carotid artery (Figure 5). Thus, embolization of the right external carotid artery was performed before surgery to reduce intraoperative bleeding and the resultant surgical difficulty. The patient then underwent hemimandibulectomy to remove the diseased tissue completely. The tumor was observed to have a mix of cystic and solid components during operation, and the histopathological examination indicated this patient’s ameloblastoma was predominantly of the plexiform type, which contained a small amount of follicular structure (Figure 6). It would be difficult to tolerate one-stage surgical repair of tissue defects due to the large amount of intraoperative blood loss (>1200 mL), low intraoperative hemoglobin level (5 mg/dL), and poor coagulation status. Iodoform gauze was used as a packing material following the resection of the giant tumor. After surgery, the patient’s serum calcium and creatinine were still stabilized within the normal range (total calcium 2.34 mmol/L, creatinine 92 µmol/L), and the patient’s facial deformity, oral function, and general condition were greatly improved (Figure 7).

**Figure 8:**
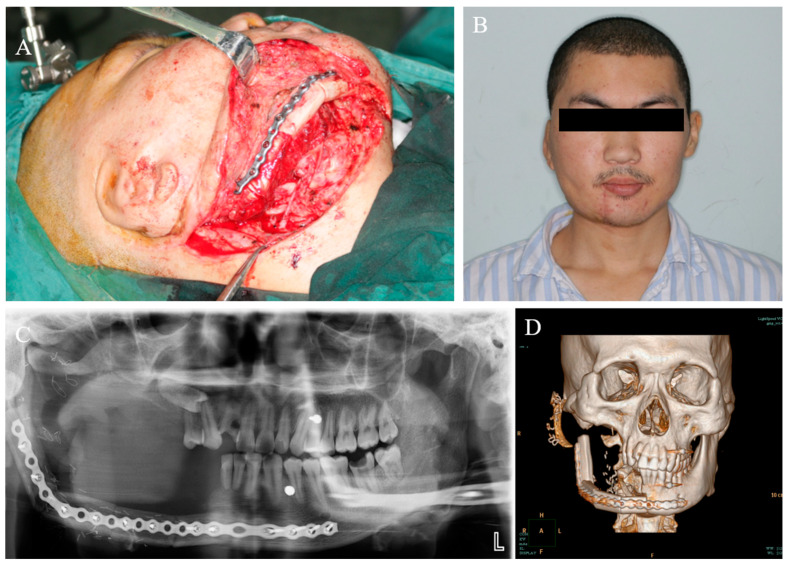
The patient received mandibular defect reconstruction with vascularized fibular myocutaneous flap. (**A**) Surgical photograph. (**B**) Clinical photograph. (**C**) Panoramic radiography. (**D**) Three-dimensional reconstruction.

Two months after the surgery, after thoughtful preoperative evaluation (total calcium 2.25 mmol/L, creatinine 81 µmol/L) when the patient was able to tolerate the second-stage surgery, he received CAM/CAD-assisted mandibular defect reconstruction with vascularized fibular myocutaneous flap. The patient had subsequent follow-ups for 5 years. His appearance and function were found to be recovering well, with a better quality of life (Figure 8). No evidence of recurrence or distant metastasis of the ameloblastoma were found by imaging examination. Additionally, blood examination confirmed no recurrence of the hypercalcemia.

## 3. Discussion

In this case study, a patient with giant ameloblastoma was found to exhibit abnormally elevated serum calcium levels during admission, which was diagnosed as hypercalcemia. As is known, when hypercalcemia is detected in the setting of malignancy, all other potential causes for the elevation of the calcium levels (e.g., hyperparathyroidism, excessive intake of calcium or vitamin D, renal failure, and the use of thiazide diuretics, among other causes) should be ruled out before considering a correlation between hypercalcemia and the tumor [9,10,11,12]. In this case, parathyroid imaging did not reveal any signs of hyperparathyroidism. Additionally, the patient presented low serum levels of PTH and vitamin D, and lacked a history of urinary disease or relevant drug usage. The above evidence suggested that the patient’s hypercalcemia was most likely attributed to the presence of a huge ameloblastoma. Furthermore, the resolution of hypercalcemia following tumor resection further emphasized the diagnosis of ameloblastoma-associated hypercalcemia.

Hypercalcemia is a frequently encountered paraneoplastic syndrome, which is often associated with an unfavorable prognosis [13]. Malignancy-associated hypercalcemia (MAH) is frequently associated with a variety of malignancies, including breast cancer, multiple myeloma, squamous-cell carcinoma, and hematopoietic malignancy [14,15]. At present, MAH can be divided into four types based on the underlying mechanisms [10,15,16,17,18]. The most common type of MAH, observed in approximately 80% of cases, is humoral hypercalcemia of malignancy (HHM) caused by the tumor’s secretion of parathyroid hormone-related protein (PTHrP). The second most common type accounting for about 20% of the cases is local osteolytic malignancy (LOH), which results from local osteoclastic bone resorption related to extensive osteolytic metastasis or primary bone malignancies. In rare cases, hypercalcemia is evoked by excess 1,25-dihydroxyvitamin D (1,25(OH)_2_D) secretion of the tumor or ectopic PTH secretion [15]. Due to the benign histological feature of ameloblastoma, there have been a limited number of cases of jaw ameloblastoma accompanied by hypercalcemia currently. According to our literature review, a total of 13 cases of ameloblastoma with hypercalcemia in the jaw (maxilla and mandible) have been reported, including the current case [19,20,21,22,23,24,25,26,27,28,29,30]. Most of these cases had local recurrence and distant metastasis (mostly to the lung), which may indicate that the ameloblastoma with a higher degree of malignancy and a more aggressive nature would be more prone to cause hypercalcemia (Table 3). Although this case was not found to have distant metastasis, the observed active bone destruction and high aggressiveness suggest that the growth pattern of this ameloblastoma was biased toward malignancy.

The availability of relevant data restricts the exploration of the mechanisms of ameloblastoma-associated hypercalcemia. To date, no osteolytic metastasis has been identified in any of the reported relevant cases. In most cases, ameloblastoma presented a marked destruction of the bone tissue, and the increased osteoclast-driven local bone resorption surrounding the tumor cells appeared to be a key cause of hypercalcemia. The local bone resorption mainly occurs through the secretion of paracrine factors by tumor cells that can stimulate osteoclast formation and bone resorption, rather than being directly attributed to tumor invasion. It has been demonstrated that ameloblastosis could cause hypercalcemia via the secretion of interleukin-1, a bone-resorptive cytokine, acting on bone tissue [31]. Another potential cause is the secretion of PTHrP by ameloblastoma cells that causes HHM. Numerous previous studies have consistently demonstrated the expression of PTHrP in the majority of ameloblastoma cases [32,33,34]. PTHrP can increase calcium reabsorption in the kidney and stimulate the secretion of receptor activator of nuclear factor-B ligand (RANKL) in osteoblasts, similar to PTH. This receptor activator binds to the corresponding receptor on osteoclasts, mediates the differentiation of osteoclast precursors into mature osteoclasts, and increases bone resorption by osteoclasts [10]. The expression of PTHrP in ameloblastoma may significantly contribute to local bone resorption, thereby elucidating the tumor’s highly aggressive and osteolytic nature [34]. The combination of the two above mechanisms may primarily lead to ameloblastoma-associated hypercalcemia. According to the literature review, including the present case, a total of five cases of benign ameloblastoma (without recurrence and distant metastasis) with hypercalcemia have been reported, all of which were giant tumors [24,26,29,30]. The large volume of ameloblastoma may lead to the increased secretion of paracrine factors and high serum PTHrP levels, which may cause severe local bone destruction. Another concern in this case is the abundant blood supply of the tumor, which may facilitate an influx of ionic calcium from osteolysis into the blood, resulting in a rapid elevation of serum calcium levels. In addition, the patient’s tumor lesion presented a localized infection (abscess formation), which has been noted in some cases of ameloblastoma with hypercalcemia. The occurrence of local infection may be a potential factor accelerating the elevation of serum calcium, as it may further contribute to the establishment of local blood circulation within the lesion and thereby promote peripheral osteolysis and elevated blood calcium.

In the case discussed in this study, abnormally elevated serum creatine levels were observed, indicating renal insufficiency. Some reported cases of ameloblastoma with hypercalcemia have also described a variety of symptoms of impaired kidney function, including elevated creatinine levels and kidney stone formation [19,20,22]. Previous studies have proposed that persistent hypercalcemia can lead to renal damage through increased vascular resistance, decreased blood volume, and the development of calcium deposits in the kidneys [35,36,37]. Elevated serum calcium levels cause renal vasoconstriction, and compromised perfusion and blood flow through the kidneys, leading to a decrease in glomerular filtration rate. In addition, increased blood calcium levels increased the excretion of calcium in the urine, while prolonged deposition of calcium–phosphorus in the kidneys can readily lead to nephrocalcinosis and/or stone formation, ultimately resulting in renal obstruction and permanent renal damage.

The patient in our study had no previous history of kidney disease prior to the occurrence of ameloblastoma, suggesting that his impaired renal function was probably related to the severe ameloblastoma-associated hypercalcemia. We observed that the patient’s serum creatinine levels dropped off along with serum calcium levels after using a range of interventions and there was no relapse of abnormal kidney function after tumor resection, which demonstrated the relationship between functional renal impairment and hypercalcemia caused by the ameloblastoma.

Instead of the reduction in the lesion, the patient exhibited tumor enlargement after marsupialization, which is another issue worth exploring. Conservative treatment methods, such as marsupialization, curettage, enucleation, cauterization, and cryotherapy, offer the advantage of maximizing the preservation of normal tissue and reduce maxillofacial deformities, while the recurrence rate after surgery is higher compared to radical treatment. The radical treatment methods emphasize the importance of achieving complete tumor resection, which commonly leads to severe facial deformity and dysfunction. Oral and maxillofacial surgeons are constantly striving to identify a treatment approach that not only reduces the recurrence of ameloblastoma but also minimizes the occurrence of facial deformity and dysfunction. In recent years, the staged surgical treatment of ameloblastoma (marsupialization in the first stage, followed by curettage or resection in the second stage) has been gradually recognized. The positive results of reducing the tumor volume and minimizing the extent of surgery by marsupialization in cystic ameloblastoma have repeatedly been reported, which can minimize the risk of operative injury [38]. Marsupialization reduces the internal pressure of the cystic cavity by interconnecting the cystic cavity with the oral cavity, which promotes the concentric shrinkage of the cyst wall and the growth of new bone. As a consequence, the majority of cystic lesions gradually decrease in size and patients may require a subsequent surgery (curettage, enucleation, or resection) or even remain untreated, depending on the outcome following marsupialization [7,8,39]. This strategy can maximally reduce surgical trauma and minimize the risk of postoperative recurrence, and also reduce the oromaxillofacial deformation and destruction. Nakamura et al. reported the efficacy of marsupialization in 31 cases with cystic ameloblastoma (including unicystic and multicystic), showing a response rate of 74.2% [40]. Zinan Yang et al. also indicated the efficacy of marsupialization in 63 patients with cystic ameloblastoma, in which the mean tumor volume decreased by 65.6% over a period of approximately 16.6 months [7]. The results demonstrated the promising therapeutic effects of marsupialization for cystic ameloblastomas, and there was no significant difference in the therapeutic efficacy of marsupialization between unicystic and multicystic ameloblastomas. However, the inconsistent efficacy of marsupialization remains a significant disadvantage, as a small proportion of patients showed no significant reduction in tumor size or even exhibited further enlargement after the procedure, ultimately necessitating extensive resection.

The efficacy of marsupialization in ameloblastomas may be related to several factors, including the growth characteristics of the tumor, the potential for new bone formation, and the technique of marsupialization [41]. The ineffectiveness of marsupialization is most likely attributed to the pathological type and local aggressiveness of ameloblastomas. Solid/multicystic ameloblastomas are relatively more aggressive and prone to invasive infiltration into adjacent tissues, which may lead to a higher recurrence rate [42]. In one study, in about half of the ineffective cases of marsupialization, the tumor had a tendency to infiltrate the surrounding tissues [41]. The “unilocular” and “multilocular” radiolucency of ameloblastomas on radiological imaging may not directly correspond to the pathological types of “unicystic” and “multicystic”, with the definite distinction requiring pathological examination results [43]. Some ameloblastomas may show wide pseudocystic spaces on the imaging, but present a histologically solid pattern, for which conservative surgery (such as marsupialization) may not be the best treatment option [38]. Although the imaging findings in this case suggested a “unilocular” appearance, the intraoperative gross observation and the histopathological examination revealed the mixed type of plexifom and follicular solid–multicystic ameloblastoma. This may explain why the tumor in this case not only failed to shrink after marsupialization but also continued to enlarge. Meanwhile, it is very important to obtain a single cystic space by marsupialization and maintain reliable connectivity with the outside world, which can effectively relieve the pressure within the cystic cavity. Confusingly, a number of studies have proposed that the marsupialization may change the type of ameloblastoma from the expansive pattern (dominated by the cuboidal cell type) to the invasive type (dominated by the columnar or basal predominant cell type) by altering the environment of the cystic lesion [41,44]. That is to say, marsupialization may have a potential to enhance tumor cell invasion and facilitate infiltration into the surrounding tissues, although more evidence is required. Consequently, patients require regular follow-up during marsupialization, and if the tumor does not shrink or even tends to enlarge, immediate surgical tumor excision should be performed. Of note, the patient in our case was not followed up with regular visits in the local hospital after marsupialization. Therefore, in the early stage of the condition, failure to promptly identify the close relationship between the deteriorating general condition and rapid tumor enlargement may result in a delay in finding the appropriate treatment options and subsequent development of severe complications. Although this staged surgical strategy is worth trying as a treatment option for young patients with ameloblastoma who find it difficult to accept radical treatment, the pathological type and patient adherence should also be taken into consideration during the choice of treatment.

Lastly, it is noteworthy that the diagnosis and treatment process of the aforementioned case involved collaborative participation from various specialized departments. The success of the patient’s surgery could not be separated from the cooperation of the MDT, in which it is emphasized that multiple professional discipline teams conduct a patient-centered discussion and formulate a reasonable individual treatment plan. The medical management of complex conditions necessitates the collaboration of multidisciplinary teams to establish standardized, individualized, and optimal comprehensive treatment protocols, which is currently the prevailing mode of clinical diagnosis and treatment [45,46]. Tumor is an extremely intricate disease, which often causes a variety of complications and presents with multi-organ involvement in the middle and late stages of pathogenesis. For patients diagnosed with a tumor, it is important to focus not only on the local treatment of tumor lesion but also consider the patients’ general conditions and other systemic diseases to perform appropriate interventions for better outcomes. The utilization of the MDT is vital in managing complex cases, as it facilitates the provision of comprehensive systemic treatment and multidisciplinary intervention, thereby contributing to an enhanced prognosis [47,48]. In this case, when the patient was admitted to our hospital, his systemic condition was found unfavorable and thus he had difficulty in tolerating the tumor resection. Fortunately, since our institution is a premier tertiary care hospital, we timely organized an MDT meeting involving the departments of radiology, neurosurgery, nephrology, anesthesiology, and endocrinology. This collaborative approach facilitated the integration of the expertise from various professional departments and offered more precise diagnosis and comprehensive treatment plan for patients. As such, several interventions were proposed and implemented for this patient who presented with ameloblastoma and multiple comorbidities, including hypercalcemia, impaired kidney function, iron deficiency anemia, and cachexia. With the aid of the MDT, the patient’s physical condition gradually recovered to a level to tolerate the surgery of the huge mandibular ameloblastoma. The good prognosis also proved that the MDT played a significant role in the diagnosis and treatment of this patient with complex conditions. When facing patients with complex conditions, oral and maxillofacial surgeons should not limit their focus of the local lesions of the head and neck region, but should also analyze and evaluate the condition of patients from a systemic perspective. More importantly, clinicians should fully utilize the superiority of institutions with comprehensive medical facilities and cooperate with other professional departments in order to provide prompt timely and professional treatment to patients, thereby fostering enhancements in medical quality.

## 4. Conclusions

Herein, we reported an unusual case of giant mandibular ameloblastoma with severe hypercalcemia and other complications including impaired kidney function, cachexia, and anemia. The hypercalcemia caused by ameloblastoma of the mandible may be related to local osteolysis and the release of PTHrP, while the exact mechanisms need to be further elucidated. While the occurrence of ameloblastoma-associated hypercalcemia is infrequent, oral and maxillofacial surgeons should also be aware of the diagnosis and treatment of this illness in clinical practice. In cases where ameloblastoma presents with substantial size or exhibits a high degree of malignancy, it is imperative to maintain a state of alertness towards hypercalcemia and promptly implement calcium-reducing measures.

Furthermore, when determining the appropriate surgical method, consideration should be given to the pathological type of ameloblastoma. The absence of ameloblastoma recurrence in this case within 5 years after surgery is most likely attributed to the appropriate choice of operation and complete tumor excision. In instances where conservative management (e.g., marsupialization) is selected, close follow-up is necessary to detect the alterations of the tumor.

It is also worth mentioning that, in the diagnosis and treatment of complex cases, the MDT can effectively bring out the best of each discipline and provide patients with a personalized treatment, which is a valuable asset that should be capitalized on by any general hospital.

## Figures and Tables

**Table 1:** Discussion results of MDT meeting and recommendations from multiple departments.

Department	Professional Recommendations
Nephrology	1. Chronic nephropathy may be associated with hypercalcemia. 2. Attention should be paid to maintaining water-electrolyte balance and acid–base balance in perioperative period. 3. If the patient’s renal function remains abnormal after surgery, further renal investigation should be considered.
Endocrinology	1. Hypercalcemia caused by hyperparathyroidism is ruled out by low parathyroid hormone levels and normal parathyroid imaging. 2. Lowering blood calcium levels to the normal range before surgery.
Radiology	CTA is recommended when necessary to clarify the relationship between the tumor and neck vessels.
Neurosurgery	If the tumor has a rich blood supply from right external carotid artery, preoperative external carotid artery embolization can be considered to prevent intraoperative bleeding.
Anesthesiology	1. The long duration of surgery, massive hemorrhage, and hemodynamic instability implies high risks of anesthesia. 2. Taking aggressive measures to optimize preoperative nutritional status. 3. Adequate supply of compatible blood and blood products should be prepared. 4. Establishing peripheral venous access before surgery.
Hematology	Low hemoglobin, low ferritin levels, and microcytic hypochromic anaemia—iron supplementation is recommended.

**Table 2:** The patient’s abnormal biochemical parameters at admission.

Biochemical Parameter	Result	Normal Values	Interpretation
Hemoglobin	91 g/L	131~172 g/L	Recurrent oral bleeding and inadequate nutrient intake may lead to anemia.
Hematokrit	28.6%	38.0~50.8%	
Total serum calcium	3.64 mmol/L	2.03~2.54 mmol/L	Hypercalcemia is likely related to the giant ameloblastoma.
Serum potassium	3.07 mmol/L	3.50~5.20 mmol/L	Deficiency of oral intake.
Serum phosphorus	0.71 mmol/L	0.87~1.45 mmol/L	Deficiency of oral intake.
Creatinine	131 umol/L	59~104 umol/L	Hypercalcemia may results in damaged renal function.
Parathyroid hormone (PTH)	6.7 pg/mL	15.0~65.0 pg/mL	Hypercalcemia may lead to secondary reduction in PTH levels.
25-hydroxyvitamin D	7.5 nmol/L	12.3~107.0 nmol/L	Hypercalcemia feedback inhibited vitamin D synthesis; insufficient intake of vitamin D.

**Table 3:** The review of reported cases of ameloblastoma associated with hypercalcemia.

Author	Publish Year	Age of Ameloblastoma	Age of Hypercalcemia	Sex	Primary Site	Tumor Size	Infection	Number of Operations	Pathological Type	Metastasis	Metastasis Site	Recurrence
Seward [19]	1975	13	27	M	Mandible	-	-	3	Metastasizing	Yes	Lungs Liver Spine	Yes
Madiedo [20]	1981	49	54	M	Maxilla	-	-	3	Metastasizing	Yes	Lungs Small intestine Peritoneal	Yes
Mcguirt [21]	1981	53	55	F	Mandible	-	-	2	Conventional	No	No	Yes
Inoue [22]	1988	51	67	F	Maxilla	-	-	2	Metastasizing	Yes	Lungs	Yes
Harada [23]	1989	33	51	M	Mandible	-	-	4	Metastasizing	Yes	Lungs	Yes
Cox [24]	2000	25	43	M	Mandible	17 × 16 × 13 cm	Yes	5	Conventional	No	No	Yes
Papaioannou [25]	2009	7	52	F	Mandible	-	-	6	Metastasizing	Yes	Lungs	Yes
Ota Y. [26]	2012	32	32	F	Mandible	272 × 203 × 151 mm	-	2	Conventional	No	No	No
Ghiam [27]	2013	26	47	M	Mandible	-	-	2	Metastasizing	Yes	Lungs	Yes
Acikgoz [28]	2014	48	52	M	Mandible	-	-	2	Metastasizing	Yes	Lungs	Yes
Lo T.E. [29]	2014	20	20	F	Mandible	12 × 12 × 8 cm	Yes	1	Conventional	No	No	No
Suzuki [30]	2020	49	49	F	Mandible	110 mm	Yes	1	Conventional	No	No	No

Age of ameloblastoma = age at diagnosis of ameloblastoma; age of hypercalcemia = age at diagnosis of hypercalcemia; M = male; F = female; - = not mentioned.

## Data Availability

Data are contained within the article.
